# 2-(4-Sulfamoylphen­yl)hydrazin-1-ium chloride

**DOI:** 10.1107/S1600536812011452

**Published:** 2012-03-21

**Authors:** Abdullah M. Asiri, Hassan M. Faidallah, Khalid A. Alamry, Seik Weng Ng, Edward R. T. Tiekink

**Affiliations:** a Chemistry Department, Faculty of Science, King Abdulaziz University, PO Box 80203, Jeddah, Saudi Arabia; b The Center of Excellence for Advanced Materials Research, King Abdulaziz University, Jeddah, PO Box 80203, Saudi Arabia; c Department of Chemistry, University of Malaya, 50603 Kuala Lumpur, Malaysia

## Abstract

The hydrazinium residue in the cation of the title salt, C_6_H_10_N_3_O_2_S^+^·Cl^−^, is twisted out of the plane of the benzene ring to which it is attached [N—N—C—C torsion angle = 25.9 (2)°] and the amino group is almost perpendicular to the benzene ring [N—S—C—C torsion angle = 88.71 (16)°]. In the crystal, the cations are linked by N—H⋯O hydrogen bonds and π–π inter­actions [ring centroid distance = 3.7280 (11) Å], forming layers in the *bc* plane that are connected by N—H⋯Cl hydrogen bonds.

## Related literature

For background to the biological applications of related sulfonamides, see: Croitoru *et al.* (2004 ▶); Dogruer *et al.* (2010 ▶). For related structures, see: Asiri *et al.* (2011 ▶, 2012 ▶).
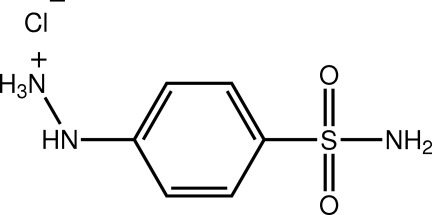


## Experimental

### 

#### Crystal data

C_6_H_10_N_3_O_2_S^+^·Cl^−^
*M* *_r_* = 223.68
Monoclinic, 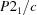

*a* = 10.2203 (8) Å
*b* = 9.8883 (7) Å
*c* = 9.1948 (8) Å
β = 107.647 (9)°
*V* = 885.51 (12) Å^3^
*Z* = 4
Mo *K*α radiation
μ = 0.64 mm^−1^
*T* = 100 K
0.35 × 0.30 × 0.25 mm

#### Data collection

Agilent SuperNova Dual diffractometer with an Atlas detector
Absorption correction: multi-scan (*CrysAlis PRO*; Agilent, 2011 ▶) *T*_min_ = 0.808, *T*_max_ = 0.857
3570 measured reflections
2026 independent reflections
1767 reflections with *I* > 2σ(*I*)
*R*_int_ = 0.024

#### Refinement

*R*[*F* ^2^ > 2σ(*F* ^2^)] = 0.031
*wR*(*F* ^2^) = 0.084
*S* = 1.03
2026 reflections
142 parameters
6 restraints
H atoms treated by a mixture of independent and constrained refinement
Δρ_max_ = 0.39 e Å^−3^
Δρ_min_ = −0.43 e Å^−3^

### 

Data collection: *CrysAlis PRO* (Agilent, 2011 ▶); cell refinement: *CrysAlis PRO*; data reduction: *CrysAlis PRO*; program(s) used to solve structure: *SHELXS97* (Sheldrick, 2008 ▶); program(s) used to refine structure: *SHELXL97* (Sheldrick, 2008 ▶); molecular graphics: *ORTEP-3* (Farrugia, 1997 ▶) and *DIAMOND* (Brandenburg, 2006 ▶); software used to prepare material for publication: *publCIF* (Westrip, 2010 ▶).

## Supplementary Material

Crystal structure: contains datablock(s) global, I. DOI: 10.1107/S1600536812011452/hb6680sup1.cif

Structure factors: contains datablock(s) I. DOI: 10.1107/S1600536812011452/hb6680Isup2.hkl

Supplementary material file. DOI: 10.1107/S1600536812011452/hb6680Isup3.cml

Additional supplementary materials:  crystallographic information; 3D view; checkCIF report

## Figures and Tables

**Table 1:** Hydrogen-bond geometry (Å, °)

*D*—H⋯*A*	*D*—H	H⋯*A*	*D*⋯*A*	*D*—H⋯*A*
N1—H1⋯Cl1	0.89 (1)	2.28 (1)	3.1319 (17)	162 (2)
N1—H2⋯O2^i^	0.88 (2)	2.03 (2)	2.835 (2)	152 (2)
N1—H3⋯Cl1^ii^	0.88 (2)	2.46 (2)	3.2136 (18)	144 (2)
N1—H3⋯O1^iii^	0.88 (2)	2.46 (2)	3.083 (2)	129 (2)
N2—H4⋯Cl1^iv^	0.89 (2)	2.67 (2)	3.3647 (16)	137 (2)
N3—H5⋯Cl1^v^	0.88 (1)	2.42 (2)	3.2656 (17)	163 (2)
N3—H6⋯Cl1^vi^	0.87 (1)	2.48 (2)	3.2467 (17)	147 (2)

Symmetry codes: (i) 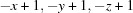; (ii) 
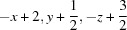
; (iii) 
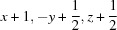
; (iv) 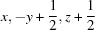; (v) 
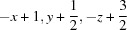
; (vi) 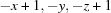.

**Fig. 1.**
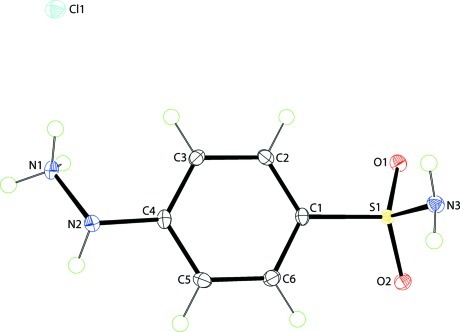
The molecular structure of (I) showing displacement ellipsoids at the 50% probability level.

**Fig. 2.**
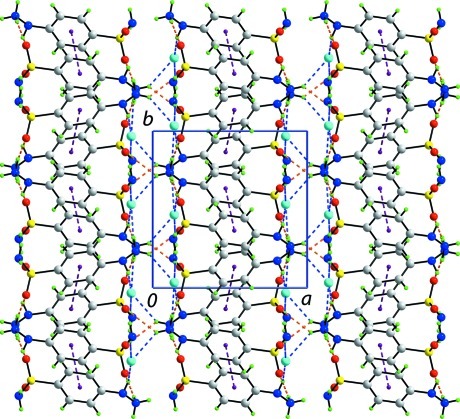
A view in projection down the c axis of the unit-cell contents of (I). The N—H···O, N—H···Cl and π—π interactions are shown as orange, blue and purple dashed lines, respectively.

## supplementary crystallographic information

### Comment

Sulphonamides related to the title salt, 2-(4-sulfamoylphenyl)hydrazinium chloride (I), are known to possess pharmacological properties. For example, *N*-substituted pyrazolyl-benzensulfonamides are known to selectively inhibit COX–2 (Croitoru *et al.*, 2004) and other derivatives were reported to exhibit anti-microbial and anti-fungal activities (Dogruer *et al.* 2010). The crystal and molecular structure of 2-(4-sulfamoylphenyl)hydrazinium chloride (I) is reported herein, as a continuation of structural studies of these systems (Asiri *et al.*, 2011; Asiri *et al.*, 2012).

The crystallographic asymmetric unit of (I) comprises a hydrazinium cation charge balanced by a chloride, Fig. 1. The hydrazinium residue is twisted out of the plane of the benzene ring to which it is attached as seen in the value of the N1—N2—C4—C3 torsion angle of 25.9 (2)°. The amino group occupies a position perpendicular to the benzene ring with the N3—S1—C1—C2 torsion angle being 88.71 (16)°; the ammonium and amino groups are orientated to opposite sides of the benzene ring.

The cations are linked by N—H···O hydrogen bonds, Table 1, and π—π interactions [ring centroid distance = 3.7280 (11) Å for symmetry operation: 1 - *x*, 1 - *y*, 1 - *z*] to form layers in the *bc* plane. The cations are connected to the chloride anions by N—H···Cl hydrogen bonds, Table 1, leading to a three-dimensional architecture.

### Experimental

Diazotization of sulfonamide with NaNO_2_/HCl followed by reduction with sodium sulfite afforded the title salt which was crystallized from ethanol as irregular light-brown chunks. Yield: 72%. *M*.pt. 488–490 K.

### Refinement

Carbon-bound H-atoms were placed in calculated positions [C—H = 0.95 Å, *U*_iso_(H) = 1.2*U*_eq_(C)] and were included in the refinement in the riding model approximation. The N—H atoms were located in a difference Fourier map, and were refined with a distance restraint of N—H = 0.88±0.01 Å; their *U*_iso_ values were refined.

### Crystal data

**Table:**

C_6_H_10_N_3_O_2_S^+^·Cl^−^	*F*(000) = 464
*M**_r_* = 223.68	*D*_x_ = 1.678 Mg m^−^^3^
Monoclinic, *P*2_1_/*c*	Mo *K*α radiation, λ = 0.71073 Å
Hall symbol: -P 2ybc	Cell parameters from 2194 reflections
*a* = 10.2203 (8) Å	θ = 2.3–27.5°
*b* = 9.8883 (7) Å	µ = 0.64 mm^−^^1^
*c* = 9.1948 (8) Å	*T* = 100 K
β = 107.647 (9)°	Irregular, light-brown
*V* = 885.51 (12) Å^3^	0.35 × 0.30 × 0.25 mm
*Z* = 4	

### Data collection

**Table:**

Agilent SuperNova Dual diffractometer with an Atlas detector	2026 independent reflections
Radiation source: SuperNova (Mo) X-ray Source	1767 reflections with *I* > 2σ(*I*)
Mirror monochromator	*R*_int_ = 0.024
Detector resolution: 10.4041 pixels mm^-1^	θ_max_ = 27.5°, θ_min_ = 2.9°
ω scan	*h* = −13→7
Absorption correction: multi-scan (*CrysAlis PRO*; Agilent, 2011)	*k* = −12→12
*T*_min_ = 0.808, *T*_max_ = 0.857	*l* = −9→11
3570 measured reflections	

### Refinement

**Table:**

Refinement on *F*^2^	Primary atom site location: structure-invariant direct methods
Least-squares matrix: full	Secondary atom site location: difference Fourier map
*R*[*F*^2^ > 2σ(*F*^2^)] = 0.031	Hydrogen site location: inferred from neighbouring sites
*wR*(*F*^2^) = 0.084	H atoms treated by a mixture of independent and constrained refinement
*S* = 1.03	*w* = 1/[σ^2^(*F*_o_^2^) + (0.0404*P*)^2^ + 0.3504*P*] where *P* = (*F*_o_^2^ + 2*F*_c_^2^)/3
2026 reflections	(Δ/σ)_max_ < 0.001
142 parameters	Δρ_max_ = 0.39 e Å^−^^3^
6 restraints	Δρ_min_ = −0.43 e Å^−^^3^

### Fractional atomic coordinates and isotropic or equivalent isotropic displacement parameters (Å^2^)

**Table:**

	*x*	*y*	*z*	*U*_iso_*/*U*_eq_	
Cl1	0.86073 (5)	−0.03338 (4)	0.61107 (5)	0.01345 (14)	
S1	0.21204 (4)	0.40936 (4)	0.48750 (5)	0.00899 (13)	
O1	0.17687 (13)	0.36536 (13)	0.33148 (14)	0.0118 (3)	
N3	0.13229 (16)	0.31234 (16)	0.57265 (18)	0.0114 (3)	
N1	0.89021 (16)	0.26661 (17)	0.72569 (19)	0.0124 (3)	
N2	0.80635 (16)	0.32732 (16)	0.80730 (17)	0.0119 (3)	
O2	0.18027 (13)	0.54566 (12)	0.51886 (15)	0.0129 (3)	
C1	0.38970 (18)	0.38531 (18)	0.5743 (2)	0.0095 (4)	
C2	0.46066 (19)	0.28593 (18)	0.5230 (2)	0.0112 (4)	
H2A	0.4148	0.2318	0.4374	0.013*	
C3	0.59980 (19)	0.26604 (18)	0.5979 (2)	0.0108 (4)	
H3A	0.6489	0.1979	0.5635	0.013*	
C4	0.66731 (18)	0.34606 (18)	0.7235 (2)	0.0094 (4)	
C5	0.59489 (19)	0.44578 (18)	0.7736 (2)	0.0119 (4)	
H5A	0.6405	0.5005	0.8588	0.014*	
C6	0.45657 (19)	0.46530 (18)	0.6994 (2)	0.0118 (4)	
H6A	0.4073	0.5333	0.7338	0.014*	
H1	0.874 (2)	0.1789 (11)	0.710 (3)	0.021 (6)*	
H2	0.879 (2)	0.302 (2)	0.6347 (16)	0.027 (7)*	
H3	0.9755 (12)	0.284 (2)	0.779 (2)	0.030 (7)*	
H4	0.842 (2)	0.4043 (16)	0.851 (3)	0.032 (7)*	
H5	0.145 (3)	0.339 (2)	0.6667 (14)	0.029 (7)*	
H6	0.148 (2)	0.2263 (11)	0.564 (3)	0.024 (6)*	

### Atomic displacement parameters (Å^2^)

**Table:**

	*U*^11^	*U*^22^	*U*^33^	*U*^12^	*U*^13^	*U*^23^
Cl1	0.0161 (2)	0.0110 (2)	0.0118 (2)	0.00202 (17)	0.00197 (18)	0.00096 (16)
S1	0.0077 (2)	0.0090 (2)	0.0098 (2)	0.00040 (16)	0.00203 (17)	0.00041 (16)
O1	0.0122 (6)	0.0137 (6)	0.0087 (6)	−0.0005 (5)	0.0018 (5)	0.0004 (5)
N3	0.0117 (8)	0.0114 (8)	0.0114 (8)	−0.0016 (6)	0.0041 (6)	−0.0003 (6)
N1	0.0074 (8)	0.0142 (8)	0.0154 (8)	0.0007 (6)	0.0030 (7)	−0.0008 (7)
N2	0.0094 (7)	0.0115 (7)	0.0133 (8)	0.0004 (6)	0.0014 (6)	−0.0020 (6)
O2	0.0119 (6)	0.0097 (6)	0.0163 (7)	0.0020 (5)	0.0029 (5)	0.0001 (5)
C1	0.0077 (8)	0.0104 (8)	0.0105 (9)	−0.0006 (7)	0.0028 (7)	0.0019 (7)
C2	0.0110 (8)	0.0108 (8)	0.0112 (9)	−0.0022 (7)	0.0025 (7)	−0.0017 (7)
C3	0.0105 (8)	0.0094 (8)	0.0135 (9)	0.0011 (7)	0.0052 (7)	0.0002 (7)
C4	0.0077 (8)	0.0096 (8)	0.0104 (8)	0.0002 (7)	0.0020 (7)	0.0043 (7)
C5	0.0136 (9)	0.0104 (8)	0.0106 (9)	−0.0011 (7)	0.0020 (7)	−0.0020 (7)
C6	0.0122 (9)	0.0111 (9)	0.0125 (9)	0.0009 (7)	0.0044 (7)	−0.0008 (7)

### Geometric parameters (Å, º)

**Table:**

S1—O2	1.4358 (13)	N2—H4	0.887 (10)
S1—O1	1.4366 (13)	C1—C2	1.386 (3)
S1—N3	1.6076 (16)	C1—C6	1.392 (3)
S1—C1	1.7640 (18)	C2—C3	1.393 (3)
N3—H5	0.876 (10)	C2—H2A	0.9500
N3—H6	0.873 (10)	C3—C4	1.397 (3)
N1—N2	1.431 (2)	C3—H3A	0.9500
N1—H1	0.886 (10)	C4—C5	1.392 (3)
N1—H2	0.883 (10)	C5—C6	1.384 (3)
N1—H3	0.877 (10)	C5—H5A	0.9500
N2—C4	1.408 (2)	C6—H6A	0.9500
			
O2—S1—O1	118.78 (8)	C2—C1—C6	120.49 (16)
O2—S1—N3	106.47 (8)	C2—C1—S1	120.96 (14)
O1—S1—N3	107.17 (8)	C6—C1—S1	118.52 (14)
O2—S1—C1	107.46 (8)	C1—C2—C3	119.51 (16)
O1—S1—C1	108.83 (8)	C1—C2—H2A	120.2
N3—S1—C1	107.66 (8)	C3—C2—H2A	120.2
S1—N3—H5	110.8 (16)	C2—C3—C4	120.19 (17)
S1—N3—H6	113.8 (16)	C2—C3—H3A	119.9
H5—N3—H6	114 (2)	C4—C3—H3A	119.9
N2—N1—H1	112.5 (15)	C5—C4—C3	119.70 (16)
N2—N1—H2	113.9 (15)	C5—C4—N2	117.49 (16)
H1—N1—H2	106 (2)	C3—C4—N2	122.76 (16)
N2—N1—H3	106.2 (16)	C6—C5—C4	120.12 (17)
H1—N1—H3	113 (2)	C6—C5—H5A	119.9
H2—N1—H3	106 (2)	C4—C5—H5A	119.9
C4—N2—N1	115.70 (14)	C5—C6—C1	120.00 (17)
C4—N2—H4	110.0 (16)	C5—C6—H6A	120.0
N1—N2—H4	111.8 (17)	C1—C6—H6A	120.0
			
O2—S1—C1—C2	−156.96 (14)	C2—C3—C4—C5	0.0 (3)
O1—S1—C1—C2	−27.13 (17)	C2—C3—C4—N2	177.52 (17)
N3—S1—C1—C2	88.71 (16)	N1—N2—C4—C5	−156.52 (16)
O2—S1—C1—C6	25.25 (17)	N1—N2—C4—C3	25.9 (2)
O1—S1—C1—C6	155.08 (14)	C3—C4—C5—C6	0.1 (3)
N3—S1—C1—C6	−89.07 (16)	N2—C4—C5—C6	−177.47 (17)
C6—C1—C2—C3	0.3 (3)	C4—C5—C6—C1	−0.1 (3)
S1—C1—C2—C3	−177.48 (14)	C2—C1—C6—C5	−0.1 (3)
C1—C2—C3—C4	−0.2 (3)	S1—C1—C6—C5	177.71 (14)

### Hydrogen-bond geometry (Å, º)

**Table:**

*D*—H···*A*	*D*—H	H···*A*	*D*···*A*	*D*—H···*A*
N1—H1···Cl1	0.89 (1)	2.28 (1)	3.1319 (17)	162 (2)
N1—H2···O2^i^	0.88 (2)	2.03 (2)	2.835 (2)	152 (2)
N1—H3···Cl1^ii^	0.88 (2)	2.46 (2)	3.2136 (18)	144 (2)
N1—H3···O1^iii^	0.88 (2)	2.46 (2)	3.083 (2)	129 (2)
N2—H4···Cl1^iv^	0.89 (2)	2.67 (2)	3.3647 (16)	137 (2)
N3—H5···Cl1^v^	0.88 (1)	2.42 (2)	3.2656 (17)	163 (2)
N3—H6···Cl1^vi^	0.87 (1)	2.48 (2)	3.2467 (17)	147 (2)

Symmetry codes: (i) −*x*+1, −*y*+1, −*z*+1; (ii) −*x*+2, *y*+1/2, −*z*+3/2; (iii) *x*+1, −*y*+1/2, *z*+1/2; (iv) *x*, −*y*+1/2, *z*+1/2; (v) −*x*+1, *y*+1/2, −*z*+3/2; (vi) −*x*+1, −*y*, −*z*+1.
